# Periodontal Ehlers–Danlos syndrome is associated with leukoencephalopathy

**DOI:** 10.1007/s10048-018-0560-x

**Published:** 2018-12-08

**Authors:** Ines Kapferer-Seebacher, Quinten Waisfisz, Sylvia Boesch, Marieke Bronk, Peter van Tintelen, Elke R. Gizewski, Rebekka Groebner, Johannes Zschocke, Marjo S. van der Knaap

**Affiliations:** 1 Department of Operative and Restorative Dentistry, Medical University of Innsbruck, Anichstr. 35, 6020 Innsbruck, Austria; 2 Department of Clinical Genetics, VU University Medical Center, De Boelelaan 1117, 1081 HV Amsterdam, The Netherlands; 3 Department of Neurology, Medical University of Innsbruck, Anichstr. 35, 6020 Innsbruck, Austria; 4 Department of Clinical Genetics, Academic Medical Center, de Boelelaan 1118, 1081 HV Amsterdam, The Netherlands; 5 Department of Neuroradiology, Medical University Innsbruck, Anichstr. 35, 6020 Innsbruck, Austria; 6 Division of Human Genetics, Medical University of Innsbruck, Peter-Mayr Str. 1, 6020 Innsbruck, Austria; 7 Department of Child Neurology and Department of Functional Genomics, Center for Neurogenomics and Cognitive Research, VU University Medical Center, De Boelelaan 1117, 1081 HV Amsterdam, The Netherlands

**Keywords:** Ehlers–Danlos, Leukoencephalopathy, Small vessel disease, Periodontitis, Complement 1

## Abstract

Here, we report brain white matter alterations in individuals clinically and genetically diagnosed with periodontal Ehlers–Danlos syndrome, a rare disease characterized by premature loss of teeth and connective tissue abnormalities. Eight individuals of two families clinically diagnosed with periodontal Ehlers–Danlos syndrome were included in the present study and underwent general physical, dental, and neurological examination. Whole exome sequencing was performed, and all patients included in the study underwent MRI of the brain. Whole exome sequencing revealed heterozygous *C1R* mutations c.926G>T (p.Cys309Phe, Family A) and c.149_150TC>AT (p.Val50Asp, Family B). All adult individuals (*n* = 7; age range 31 to 68 years) investigated by MRI had brain white matter abnormalities. The MRI of one investigated child aged 8 years was normal. The MRI pattern was suggestive of an underlying small vessel disease that is progressive with age. As observed in other leukoencephalopathies related to microangiopathies, the extent of the white matter changes was disproportionate to the neurologic features. Medical history revealed recurrent headaches or depression in some cases. Neurological examination was unremarkable in all individuals but one had mild cognitive decline and ataxia and experienced a seizure. The observation that periodontal Ehlers–Danlos syndrome caused by missense mutations in *C1R* is consistently associated with a leukoencephalopathy opens a new pathogenic link between the classical complement pathway, connective tissue, brain small vessels, and brain white matter abnormalities.

## Introduction

Periodontal Ehlers–Danlos syndrome (EDS) is a specific EDS subtype characterized by premature loss of teeth due to severe periodontitis, increased rate of infections, and connective tissue abnormalities like easy bruising, pretibial discolorations, joint hypermobility, and organ or vessel rupture. Periodontal EDS is caused by heterozygous mutations in *C1R* or *C1S* [1], which encode subunits C1r and C1s of the first component of the classical complement pathway. This pathway is central in microbial–host interactions. Its over-activation or deregulation may excessively amplify inflammation. Additionally, the C1q subunit—binding partner of the C1s-C1r tetramer—has a triple helical structure, which resembles collagen; some features of periodontal EDS may be due to abnormal C1r/C1s interaction with extracellular matrix components [1]. For example, interactions of C1q with bone morphogenetic protein-1 and tolloid-like proteinases, metalloproteinases that have major roles in extracellular matrix assembly, have recently been shown [2].

The different EDS subtypes are not usually associated with neurological symptomatology; brain MRI is typically unremarkable [3]. There are single reports on polymicrogyria [4–7], corpus callosum agenesis or hypoplasia [7, 8] periventricular heterotopia, ventricular dilatation or cerebral atrophy [7, 9] in hypermobile, classical, or unspecified EDS. Bi-allelic mutations in *COL3A1*, encoding type III (pro) collagen, cause cobblestone-like cortical malformation and white matter changes [10–12], but these are not observed in vascular EDS caused by heterozygous *COL3A1* mutations. Leukoencephalopathy has been reported in a single individual with typical clinical features of periodontal EDS prior to identification of the causative gene defect [13]. Here, we report that leukoencephalopathy appears to be a general feature of periodontal EDS caused by *C1R* mutations.

## Methods

### Clinical investigations

Individuals of two families clinically diagnosed with periodontal EDS were included in the study. All patients included in the study underwent general physical and neurological examination. The clinical investigation list and the questionnaire for reporting on periodontal EDS were previously published in Kapferer-Seebacher et al. [14] as supplements. Briefly, clinical investigation included assessment of joint and skin features and photographs. Oral investigation included periodontal investigations, orthopantomogram, and intraoral photographs. The diagnosis of early severe periodontitis was based on radiologic evidence of severe alveolar bone loss (≥ 50%) at an age of ≤ 25 years or history of complete tooth loss due to tooth mobility at an age of ≤ 30 years. The questionnaire includes questions on previous dental treatments and smoking, joint and skin features, and other features like recurrent infections, hoarse voice, organ ruptures, and other systemic conditions. Family A was investigated at the VU University Medical Center, Amsterdam, in 2017. Clinical and genetic data of Family B have been previously reported by Kapferer-Seebacher et al. [1]. Neurologic investigation of family B was conducted in 2017 and 2018 at the Medical University of Innsbruck, Austria.

### Magnetic resonance imaging

All patients included in the study underwent MRI of the brain. We scored all MRIs according to a previously published standardized protocol [15].

### Genomic analysis

Whole exome sequencing was performed on three affected individuals from family A. Genomic DNA was isolated from blood and 2.5 μg was sheared on a Covaris S2 instrument (Covaris, Woburn, MA). DNA libraries were prepared using Kapa Biosystems reagents (Kapa Biosystems, Wilmington, MA), and 1.0 μg of library was used for enrichment with Roche/NimbleGen SeqCap EZ MedExome (Roche, Basel, Switzerland) according to the manufacturer’s protocol. Sequencing was performed on an Illumina HiSeq 2500 platform (Illumina, San Diego, CA, USA) with 125 base pair paired-end reads. Over 92% of the capture region was covered ≥ 30× with a mean bait coverage of approximately 105× for each sample. Variant calling was performed using an in-house analysis pipeline. Alignment of sequence reads to the human genome (hg19) was performed with the Burrows–Wheeler Aligner tool (BWA-MEM v0.7.10) using default settings. Subsequently, Picard Tools (v1.111; http://picard.sourceforge.net/) was used for sorting and marking duplicates. For local realignment and base quality score recalibration, we used the Genome Analysis Toolkit (GATK; v3.3-0; Broad Institute, Cambridge, MA) and for variant calling, we used the GATK HaplotypeCaller. Variants were filter tagged using the GATK VariantFiltration and annotated by snpEff (v4.0). Variant prioritization was performed using Cartagenia Bench Lab NGS (Agilent Technologies, Santa Clara, USA). In short, a classification tree was used to select for variants present in all three affected individuals and virtually absent in control cohorts dbSNP build 142 (http://www.ncbi.nlm.nih.gov/projects/SNP), 1000 Genomes Phase 3 release v5.20130502, and ESP6500 (http://evs.gs.washington.edu/EVS/) as well as in-house controls. Prerequisite was that these variants had been genotyped in at least 200 alleles. Subsequently, the remaining variants were further prioritized based on literature, predicted (deleterious) effects on protein function by, e.g., truncating the protein, affecting splicing, amino acid change, and evolutionary conservation.

## Results

### Subjects, families and genetic results

**Fig. 1:**
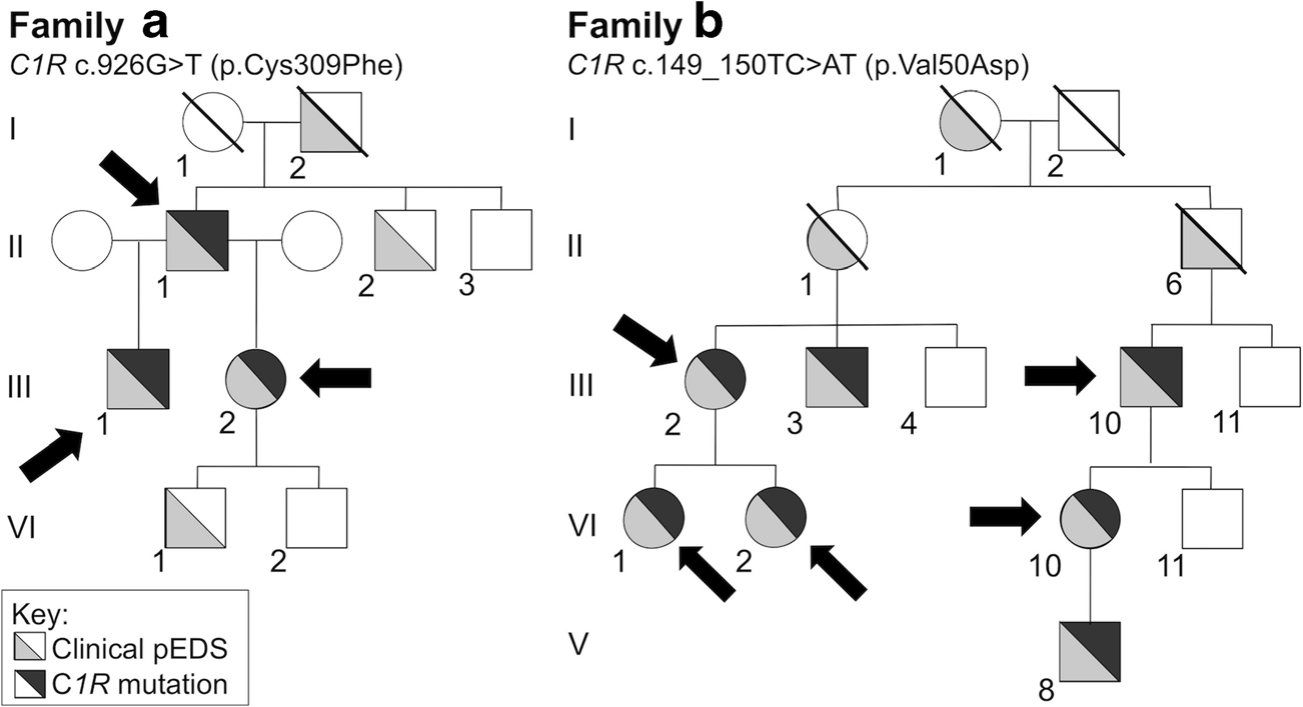
Pedigrees of the families described in this study. Above each pedigree, the *C1R* genotype in the affected individuals is specified on nucleotide and predicted protein levels. The whole pedigree of family B has been published elsewhere; identification codes are identical with Kapferer-Seebacher et al. [1]. The arrows indicate the individuals who were available for neurological examination. All indicated individuals were investigated by MRI and had brain white matter abnormalities, except individual A:III-1 at age 7 years

**Fig. 2:**
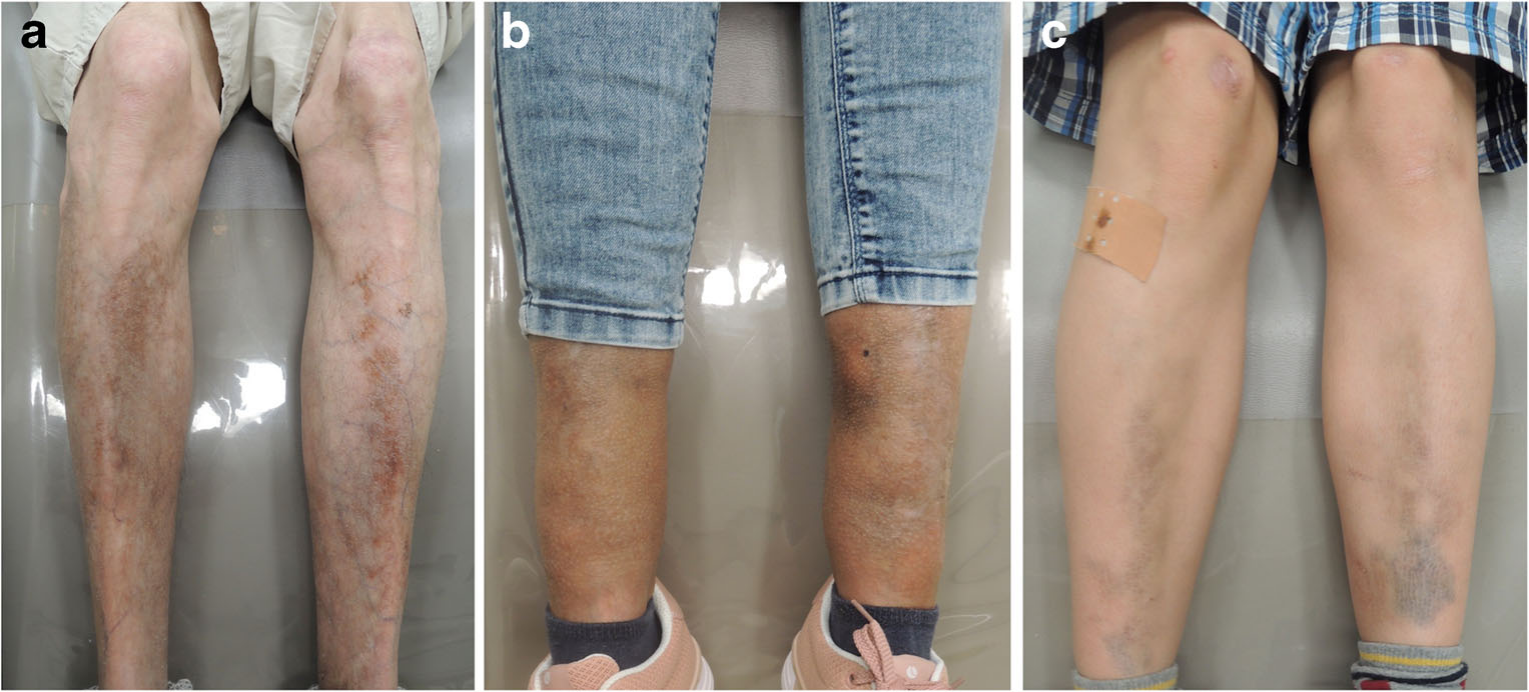
Pretibial discolorations in family A. Pretibial hemosiderotic plaques have been discribed in 83% of individuals affected with periodontal EDS (Kapferer-Seebacher et al. [1]). Photographs of shins (**a**) individual A-II-1, (**b**) individual A-III-2, and (**c**) individual A-III-1. No pretibial plaques were present in family B

*Family A* is a Caucasian Dutch four-generation family (Fig. 1); individuals A-II-1, A-III-1, and A-III-2 were available for clinical and genetic investigations. The *C1R* mutation c.926G>T (NM_001733.4) resulting in p.(Cys309Phe) was identified in all of them. This cysteine at position 309 is located in the Sushi/SCR/CCP domain and is involved in disulfide bond formation that stabilizes the C1r CCP1 module [1]. In addition, c.927C>G results in an amino acid change of the same amino acid p.(Cys309Trp) and was previously identified in other periodontal EDS patients [1]. No known or likely pathogenic variants were found in 154 genes related to leukoencephalopathies, including vascular leukoencephalopathies*.* All individuals were clinically diagnosed with periodontal EDS (for details see, Table 1; Figs. 1 and 2).

**Table 1:** Clinical features and MRI findings in individuals with periodontal EDS

Individual	Gender	Age (years)	Clinical features suggestive of periodontal EDS	Neurologic features	MRI findings
A:II-1	m	57	Early severe periodontitis; complete tooth loss at age 19 years prominent vasculature (palate); easy bruising; pretibial hemosiderotic plaques; chord stenosis; rupture of the lung and diaphragm; scoliosis; inguinal hernia; frequent respiratory tract infections	Slow and mild cognitive decline, mild tremor, slight cerebellar ataxia	Diffuse, homogeneous cerebral white matter signal abnormalities; enlarged perivascular spaces; lacunar infarcts; a few microbleeds in the basal and dentate nuclei
A:III-1	m	8	Severe gingival inflammation; lack of attached gingiva; easy bruising especially in the face; pretibial hemosiderotic plaques;	Mild learning problems	Normal
A:III-2	f	31	Early severe periodontitis; first tooth loss at age 14y; severe gingival recession; lack of attached gingiva; easy bruising; pretibial hemosiderotic plaques; slow wound healing; frequent hematomas on the thighs, e.g., after hot shower	None	White matter signal abnormalities of limited extent
B:III-2	f	56	Early severe periodontitis; complete tooth loss at age 35 years; easy bruising; mild skin hyperelasticity	None	Small and larger spots of white matter signal abnormalities; enlarged perivascular spaces
B:III-10	m	68	Complete tooth loss at age 18 years; easy bruising; mild skin hyperelasticity; inguinal hernia; organ ruptures	None	Slight generalized cerebral atrophy; extensive, homogeneous cerebral white matter signal abnormalities; enlarged perivascular spaces
B:IV-1	f	35	Early severe periodontitis; severe gingival recession; lack of attached gingiva; easy bruising; joint hypermobility of the digits; mild skin hyperelasticity	Depression	A few small spots of white matter signal abnormalities
B:IV-2	f	34	Early severe periodontitis; gingival recession; lack of attached gingiva; easy bruising; joint hypermobility of the digits and the elbows; scoliosis	Frequent fainting spells and dizziness; frequent headaches	Multiple small focal lesions in the deep cerebral white matter; enlarged perivascular spaces
B:IV-10	f	43	Early severe periodontitis; severe gingival recession; lack of attached gingiva; mild skin hyperelasticity	None	Small and larger spots of white matter signal abnormalities; enlarged perivascular spaces

*EDS* Ehlers–Danlos syndrome, *MRI* magnetic resonance imaging, *m* male, *f* female

At clinical investigation, the male *individual A:II-1* was 58 years of age. In addition to signs of periodontal EDS, his medical history included diabetes type II, high blood pressure, hypercholesterolemia, and cardiac arrhythmias; he never smoked. From age 50 years he experienced slow and mild cognitive decline with memory, concentration and spatial orientation problems, dyspraxia, aggression, and depression. At age 57 years, he was admitted in a state of confusion during pneumonia, while in the hospital, he had a seizure lasting 20 min with a focal start in the right hand and secondary generalization. Neurological examination at age 57 years revealed mild tremor and slight cerebellar ataxia, no other abnormalities. *Individual A:III-2* was 31 years of age at time of investigation. She never smoked, had a normal blood pressure, and had no complaints of cognitive decline. Neurological examination at age 31 years was normal. *Individual A:III-1* was 8 years of age at time of investigation. He had mild learning problems, especially language problems, but followed normal level education. Neurological examination at age 8 years was normal.

**Fig. 3:**
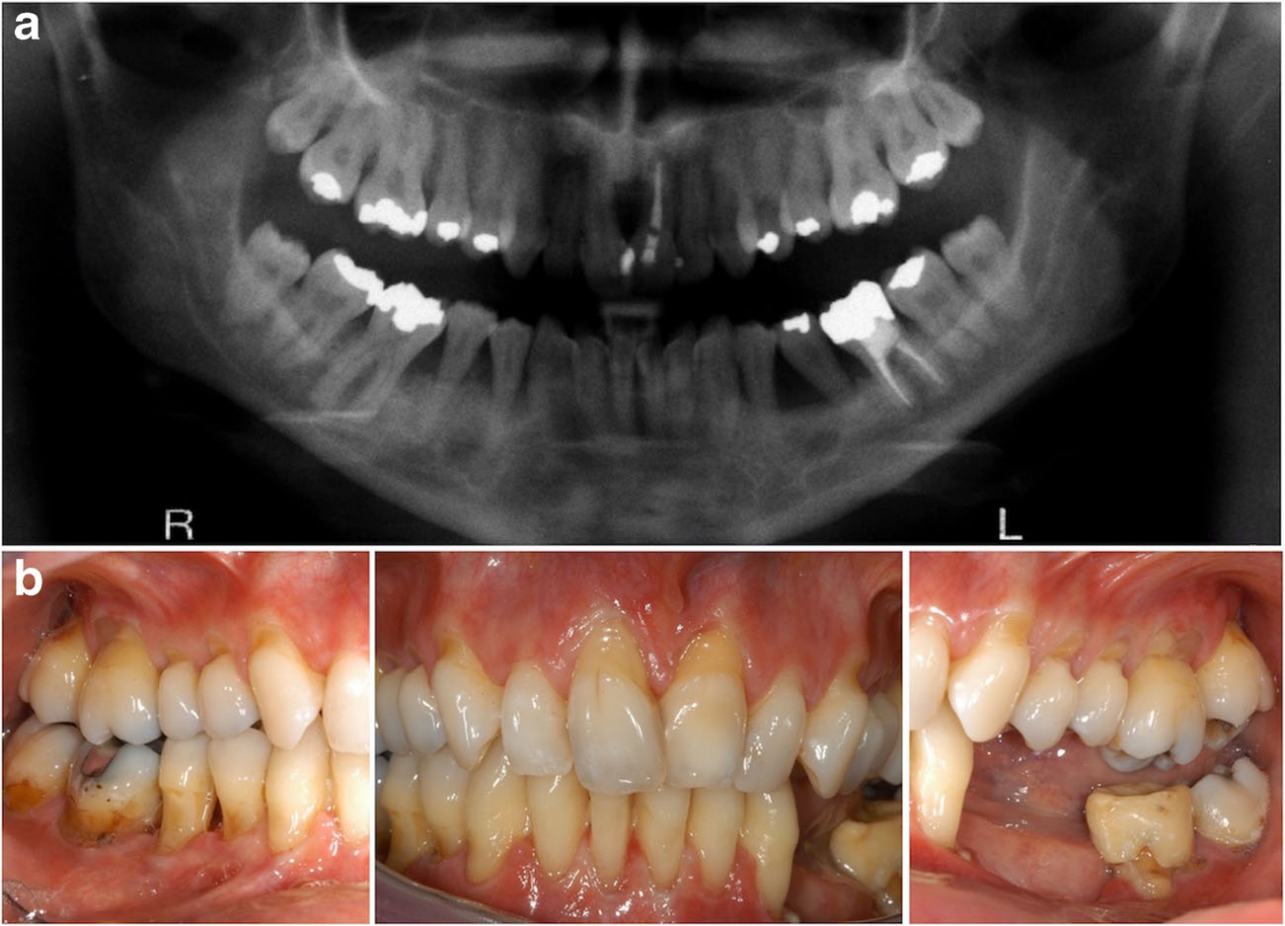
Oral features. **a** Intraoral radiograph of individual B:IV-10 at age 32 years. Notice severe periodontal bone loss in the lower jaw, especially teeth no. 44, 45, and 34, 35. **b** Intraoral photographs at age 34 years. Teeth no. 34 to 36 have been lost. Severe gingival recession (exposed tooth roots) and lack of attached gingiva with mucosa extending to the gingival margins is specific for periodontal EDS

*Family B* is a five-generation Caucasian Austrian family, whose clinical and genetic data were previously reported (Family 1 in Kapferer-Seebacher et al.) [1]. The identified *C1R* mutation c.149_150TC>AT (p.Val50Asp) segregated with 15 individuals clinically affected by periodontal EDS in this family; in Fig. 1, only individuals relevant for the present study are depicted, with the same numbers as used in Kapferer-Seebacher et al. [1]. Individuals B:III-2, B:III-10, B:IV-1, B:IV-2, and B:IV-10 were available for the present neurologic analysis. All investigated individuals of family B had never smoked or were light smokers (≤ 5 cigarettes per day), and had no hypertension or hypercholesterinemia. All affected individuals in this family showed normal intellectual performance without evidence of cognitive decline (Fig. 3).

In male *individual B:III-10*, neurological examination at age 68 years was normal. The female *individual B:III-2* reported on frequent headaches; neurological examination at age 56 years was normal. In female *individual B:IV-10*, neurological examination at age 43 years was normal. The female *individual B:IV-1* suffered from depression. Neurological examination at age 34 years was normal. The female *individual B:IV-2* was not available for clinical neurologic investigation, but reported frequent fainting spells and dizziness, and a history of frequent headaches in childhood.

### Magnetic resonance imaging

**Fig. 4:**
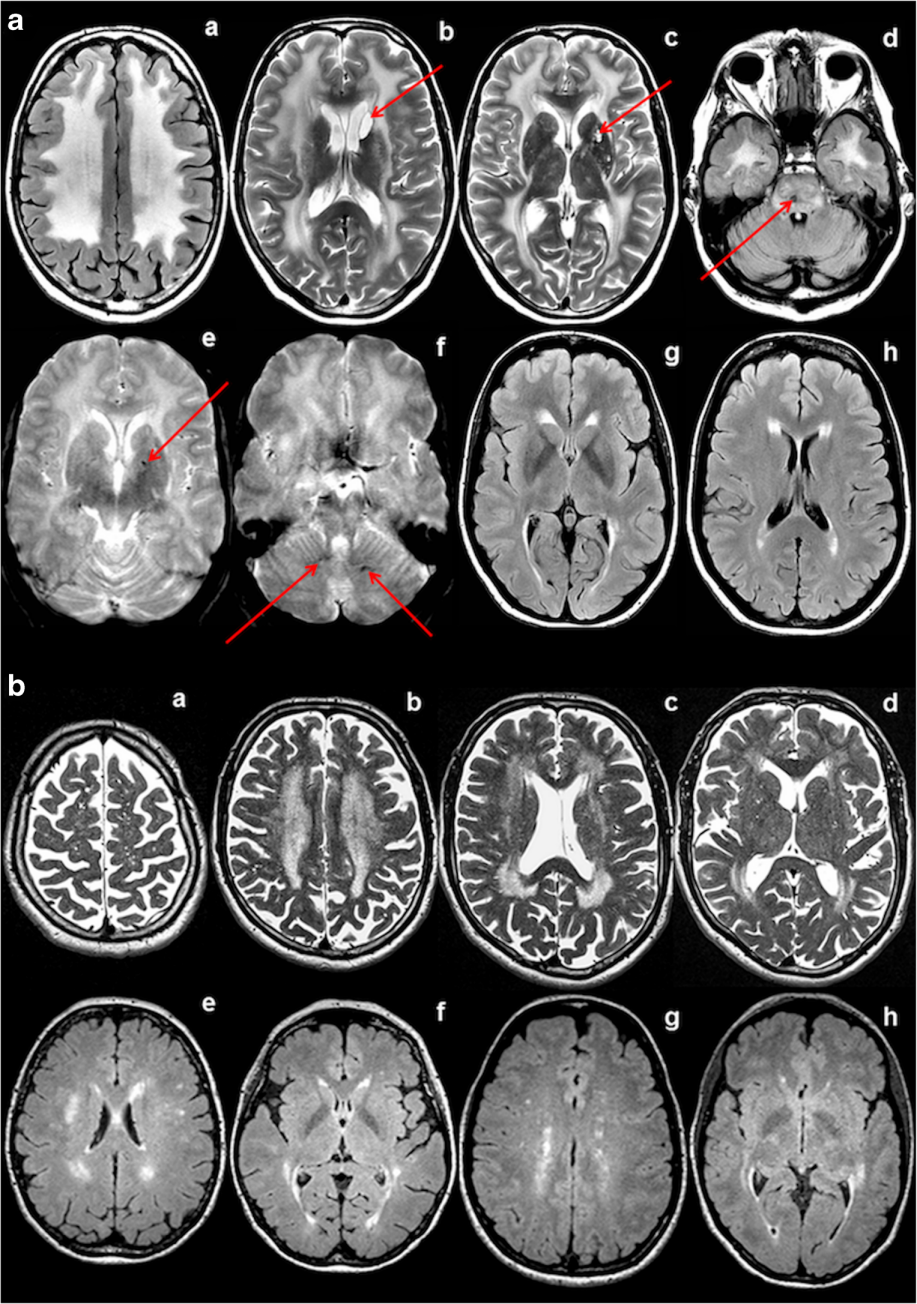
MRI. **a** Family A, (a–f) individual A:II-1 at age 57 years; (g, h) individual A:III-2 at age 31 years. The FLAIR (a, d) and T2-weighted (b, c) images in the older patient show virtually diffuse cerebral white matter signal abnormalities, including also the external and extreme capsules (c) and the anterior temporal white matter (d). Lacunar infarcts are seen in the head of the caudate nucleus on the left (arrow in b), left putamen (arrow in c), and pons (arrow in d). Numerous small areas of abnormal signal are seen in the basal ganglia and thalami (c) and the pons (d). Gradient echo images show microbleeds in the basal nuclei and dentate nucleus (arrows in e and f). The FLAIR (g, h) images in the younger patient show a thin periventricular rim as well as a few larger areas of abnormal signal in the deep cerebral white matter. **b** Family B, (a–d) individual B:III-10 at age 68 years (e, f); individual B:III-2 at age 56 years and (g, h) individual B:IV-10 at 43 years. In the oldest patient, the T2-weighted images show extensive and confluent periventricular and deep cerebral white matter abnormalities (a–d), as well as numerous enlarged perivascular spaces in the white matter (a–c) and basal nuclei (d). There is a mild generalized atrophy. The FLAIR images in the younger patients (e–h) show a thin periventricular rim as well as multifocal small and lager larger areas of abnormal signal in the deep cerebral white matter

In *family A*, *individual A:II-1* underwent MRI at age 57 years (Fig. 4a (a–f)). It showed no cerebral or cerebellar atrophy. Almost diffuse, homogeneous cerebral white matter signal abnormalities were present, with an anterior-posterior gradient, in which the posterior cerebral white matter was somewhat better preserved. A thin rim of directly subcortical white matter was relatively, but not entirely spared. The anterior part of the temporal lobe and external and extreme capsules were affected. The corpus callosum and internal capsule were spared. Within the basal nuclei and thalami numerous small foci of high T2 signal were seen, which in part had a low signal on FLAIR, indicative of enlarged perivascular spaces. Two foci in the putamen and head of the caudate nucleus on the left were larger and cystic, indicative of lacunar infarctions. Within the brain stem, a lacunar infarction was present in the pons. Inhomogeneous T2 hyperintensities were present in the pons, the lower part of the midbrain and the cerebellar white matter. Gradient echo images showed evidence of a few microbleeds in the basal nuclei on the left and the dentate nucleus on both sides. No areas of diffusion restriction or abnormal contrast enhancement were seen. *Individual A:III-2* underwent MRI at 31 years (Fig. 4a (g and h)). It showed signal abnormalities in the deep parietal and periventricular frontal and occipital white matter of limited extent. No signal abnormalities elsewhere, enlarged perivascular spaces, lacunar infarcts, areas of diffusion restriction, or microbleeds were visible. *Individual A:III-1* underwent MRI at 7 years. It was normal.

In *family B*, *individual B:III-10* underwent MRI at age 68 years (Fig. 4b (a–d)). It showed slight generalized cerebral atrophy with enlarged lateral ventricles and subarachnoid spaces. Extensive, homogeneous cerebral white matter signal abnormalities were present, without anterior–posterior gradient. The subcortical white matter was preserved in all areas and the anterior part of the temporal lobe and external and extreme capsules were not affected. The corpus callosum, internal capsule, brain stem, and cerebellum were spared. Numerous enlarged perivascular spaces were present spread over the cerebral hemispheres, involving white matter, basal nuclei and thalami. No lacunar infarcts, areas of diffusion restriction or abnormal enhancement after contrast were present. MR angiography and venography did not reveal vessel abnormalities. *Individual B:III-2* underwent MRI at age 56 years. It showed numerous enlarged perivascular spaces. FLAIR showed a thin, continuous periventricular rim of abnormal signal and numerous small and larger spots of abnormal signal spread over the deep cerebral white matter. No lacunar infarcts, areas of diffusion restriction, or abnormal contrast enhancement were present. MR angiography and venography did not reveal vessel abnormalities. *Individual B:IV-10* underwent MRI at age 43 years. The abnormalities were similar to those of individual B:III-2, but less extensive. *Individual B:IV-1* underwent MRI at age 35 years. It only showed a very thin, continuous periventricular rim and a few small spots of abnormal signal spread over the deep cerebral white matter on FLAIR. MRI of *individual B:IV-2* was obtained at age 33 years because of the neurological complaints described above. It revealed numerous enlarged perivascular spaces in the cerebral white matter. Additionally, a thin, continuous periventricular rim of abnormal signal and multiple small focal lesions were present in the deep cerebral white matter. No areas of diffusion restriction, microbleeds or abnormal contrast enhancement were observed.

## Discussion

We report two families with eight individuals clinically and genetically diagnosed with autosomal-dominant periodontal EDS who underwent MRI of the brain. In both families, periodontal EDS is associated with an adult-onset leukoencephalopathy. In young-adult patients, MRI shows multifocal signal changes in the deep cerebral white matter and enlarged perivascular spaces. In older patients, the leukoencephalopathy has become extensive to virtually diffuse and additional lesions and lacunar infarctions are present in the basal nuclei, thalami, pons, and cerebellum. One older patient has microbleeds. The latter MRI pattern is suggestive of an underlying small vessel disease [16]. Most common risk factors for leukoencephalopathies caused by small vessel disease are hypertension, hypercholesterolemia, diabetes mellitus, and age [17, 18]. Genetic vascular leukoencephalopathies, including CADASIL [19], CARASIL [20], CARASAL [21], and *COL4A1-* or *COL4A2-*related disorders in adults [22, 23], cause a similar MRI pattern. With the anterior temporal white matter abnormalities and involvement of the external and extreme capsules, the MRI pattern of our EDS patients is in fact very similar to that of CADASIL [20].

Leukoencephalopathy has been previously reported in a single individual diagnosed with periodontal EDS [13]. Unfortunately, the patient is not available for clinical follow-up and genetic confirmation of the diagnosis, but the reported clinical manifestations including pretibial skin discoloration are pathognomonic for periodontal EDS. Considering the consistent association of a vascular leukoencephalopathy with periodontal EDS in three families, it is likely that it is causally related to periodontal EDS. Neurologic features in the patient reported by Spranger et al. [13] were limited to recurrent headaches and several episodes of drop-attacks without loss of consciousness or other neurologic deficits; intellectual performance was reported normal. Also in our patients, neurological deficits are mild or absent. As in other small vessel diseases, the leukoencephalopathy in periodontal EDS is disproportionate to the severity of the clinical disease [21, 24]. Individual A:II-1 is the only patient, who presented with neurological complaints. He also is the only one with multiple vascular risk factors, including diabetes type II, hypertension, and hypercholesterolemia, which might have contributed to the disease.

The pathogenesis of the leukoencephalopathy caused by *C1R* mutations in periodontal EDS is unclear. All mutations identified so far appear to cause periodontal EDS through a gain of function effect; none of the mutations identified to date alter the enzymatic region of the molecules. There is evidence that the mutations cause intracellular retention of C1r/C1s protein and expansion of the endoplasmatic reticulum which may interfere with processing of collagen and/or other components of the exctracellular matrix (ECM) [1]. In addition, abnormal activation of the complement 1 subunits causing intracellular and extracellular presence of serine protease may alter ECM matrix proteins and trigger an immune response that could lead to arteriolar damage and white matte changes as observed in other microangiopathies. No brain autopsy data are available from affected individuals, but histopathologic investigation of urticaria1 lesion in one patient revealed small-vessel vasculitis consisting of neutrophils, mononuclear cells, and endothelial disruption (leukocytoclastic vasculitis) [25]. Capillary fragility plays a major role in periodontal EDS, and easy bruising is a major criterion of periodontal EDS [26]. However, this is also found in other forms of EDS without MRI abnormalities, and there is no evidence that vascular connective tissue fragility is the primary cause of the apparently insidious development of leukoencephalopathy in pEDS.

In conclusion, leukoencephalopathy appears to be part of the clinical spectrum in periodontal EDS with *C1R* mutations, as it was evident in all adult individuals studied by MRI known to us. The MRI pattern is suggestive of an underlying small vessel disease that progresses with age. The extent of MRI changes is disproportionate to the presence of neurologic disease features. Longitudinal studies and histopathological analyses in patients with periodontal EDS will have to elucidate the exact cause and neurological consequences of these findings.
